# Luxury or normal goods? Evidence from the utilization of institutional care services for the disabled older adults in China

**DOI:** 10.3389/fpubh.2023.1289502

**Published:** 2024-01-05

**Authors:** Xueqing Xia, Quanlun Li

**Affiliations:** School of Public Administration, Zhongnan University of Economics and Law, Wuhan, Hubei, China

**Keywords:** pension benefits, urban disabled older adult, institutional care service, Normal goods, burden on children

## Abstract

**Background:**

Nursing care is essential for older adults with disabilities. Income plays a crucial role in determining the utilization of institutional care services. Pension benefit, as the main source of income for the older adults in China’s cities and towns in their later years, is an important factor influencing the utilization of institutional care services. However, there have been no consistent findings on how pension benefits affect the utilization of institutional care services for the disabled older adults.

**Methods:**

This paper utilizes data from the 2017–2018 Chinese Longitudinal Healthy Longevity Survey. We select disabled older adults aged 65 and older, living in towns and cities, and use a probit regression model to investigate the impact of pension benefits on the utilization of institutional care services by urban disabled older adults empirically.

**Results:**

The study shows that a 1% increase in pension benefits raises the probability that the urban disabled older adults use institutional care services by 0.03. It also finds that for low-income urban disabled older adults, the effect is statistically significantly positive at the 1% level; but for high-income urban disabled older adults, the effect is not statistically significant. The pension benefits significantly increase the probability for the disabled older adults who are male, financially dependent, and live in townships. In addition, the pension benefits significantly reduce the probability that children will provide care and pay for care services for their older parents.

**Conclusion:**

Institutional care service is a normal good for the urban disabled older adults, especially for low-income older adults. Therefore, higher pension benefit raises the probability of utilizing institutional care services for the urban older adults with disabilities, and this positive effect is especially pronounced for older adults who are male, financially dependent, and reside in townships. In addition, increase in the pension benefits for the disabled older adults in towns and cities reduces the burden on children by reducing the probability that children will provide care and pay for care services for the older adults.

## 1 Introduction

The Report on the Demand for Older Adult Care Services in China’s Cities issued in 2022 points out that the new era of older adult care in cities is characterized by a “three-stage” approach to old-age care: at the vitality stage and the semi-disabled stage, home and community care is the mainstay, but at the disabled stage, more than half older adults choose to stay in specialized care institutions. Under the dual factors of aging and childlessness, there are more and more older adults living alone in cities. Urban residents prefer specialized institutions to obtain care services. With the decline of physical function, more than half of urban residents will choose to stay in institutions. According to the data released by China National Committee on Aging, in 2020, there will be more than 42 million disabled older adults over the age of 60 in China, accounting for about 16.6% of the total older adult population. Facing such a huge group of disabled older adults, institutional care services face a huge potential demand.

Studies have shown that the use of care services has a “Pro high-income” characteristic. The higher the income level of the older adults, the higher the utilization rate of care services (1, 2). The income level of an older adult directly affects whether the older adult can receive institutional care services (3). With the rapid development of China’s economy and the continuous improvement of the social security system, the income level of the older adults is increasing. According to the 2011–2018 China Health and Retirement Longitudinal Study, the annual *per capita* income of China’s urban older adults has increased from 21,135 yuan to 38,645 yuan and pension accounts for about 79% of the total income. Pension has become the first source of income for the urban older adults. However, research from the United States has found that an increase in social security income can lead to the substitution of formal home care services for institutional care services, leading to a decrease in the utilization of institutional care services (4). It can be seen that the research conclusions on the impact of pension benefits on the utilization of institutional care services are still inconsistent. In China, there is a significant difference in the supply of care institutions and income level between urban and rural areas. In urban areas, the quantity and quality of nusing care institutions are far superior to those in rural areas (5). And the income level of urban older adults is higher, 4.5 times that of rural older adults (6). Therefore, for urban older adults, income determines their ability to pay for institutional care services. Based on the Chinese background, this paper studies the impact of pension benefits on the utilization of care services in urban disabled older adults in China. Simultaneously attempting to explore the following issues: Is there heterogeneity in the effect of pension benefits on the utilization of institutional care services for different types of disabled older adults? Whether the pension benefits have eased the burden on the children who supports the older adults?

Using data from the Chinese Longitudinal Healthy Longevity Survey 2017–2018 (CLHLS), on the basis of a family decision model for older adults (7), this paper finds that an increase in pension significantly increases the probability of using institutional care services for the disabled older adults in cities and towns. The pension benefits increase by 1%, the probability of using institutional care services increases by 0.03. For the urban disabled older adults, institutional care services are normal goods, so the demand for institutional care services expands with the increasing pension benefits. In particular, the effect is more significant for disabled older persons who are male, financially dependent, and live in townships. The increased pension benefit reduces the probability of children providing home care and paying for care services, which in turn reduces the burden on children.

The contributions of this paper are as follows: (a) This paper enriches empirical research that pension benefits affect the utilization of institutional care services for the disabled older adults; (b) This study provides empirical evidence that institutional care service is a normal good for disabled older adults; (c) We find differences in the impact of pension benefits on the utilization of institutional care services by the disabled older adults at different income levels, which provides a more detailed reference for the formulation of public service policies for the older adults.

The remainder of this paper is organized as follows. Part II is the literature review and theoretical analysis; Part III is the data and model; Part IV is the empirical results, including benchmark regression results, robustness test and heterogeneity analysis; Part V is the discussions. The last part is the conclusions.

## 2 Literature review and theoretical analysis

### 2.1 Literature review

A large body of literature has focused on the factors that influence older adults’ utilization of the institutional care services. The quality of the care and distance were to be seem the most important consideration (8). However, older adults may make choice based on the high hotel services quality but not clinical care quality (9). Price also seems to has a negative impact on nursing home choice whereas the reported quality did not have an effect (10). The older adult’s age and disability, were proved to be the main determinants of utilization (11). A study in Taiwan showed that the choice of nursing home care for older people to be a process of forced choice, due to the lack of home care (12). Government policies may also have an impact on the utilization of institutional care services. Older adults enrolled in both Medicare and Medicaid, are 9.7 percentage points more likely to be admitted to a low quality institution (13). In Korean, a study found that the subsidized older adults who used the long-term care, were more likely to be low-income, female, and living alone and they were more likely to choose institutionalized care (14). Income and utilization of institutional care for the older adults are significantly correlated. Evidence from Spain showed that formal services are concentrated among the better-off, while intensive informal care is concentrated among the worst-off (15). Study from Japan found that home help services were used more by lower income households, while daycare services were used more by their higher income counterparts (16). Exploiting the exogenous variation resulting from the value of housing assets during the Great Recession, a study examined the effect of housing wealth on use of nursing home care by older adults, and found that the bursting of the housing bubble reduced nursing home entry by 1%, which was about a 30% reduction in the probability of using nursing home care (17).

Previous studies have found that there is a positive relationship between social security income and the use of formal care. Based on the sample of disabled older adults, Kemper found that the use of formal care by disabled older adults increased as their income increased (18). Wang (19), Tsai (20), and Steinbeisser et al. (21) found that with the increase in social security income, the older adult’s use of formal care services increased. However, Ettner used data from the National Long-Term Care Survey and found that there was no statistical correlation between income and the use of long-term care, but it was positively correlated with formal family care (22). Peng et al. found that economic income has a significant “U-shaped” effect on the choice of formal long-term care mode, with increasing income having no effect on the utilization of long-term care services for low-income older adults, but significantly increasing the probability of long-term care services for high-income groups (23). Meanwhile, for those older adults who had never used formal care services, higher social security income increased the likelihood of using formal care services (20). The above research focuses on formal care and long-term care, and institutional care services are only a part of them. The conclusion that income affects the utilization of institutional care services is controversial. Goda et al. divided long-term care into home care services and institutional care services when studying the impact of pension benefits on the utilization of long-term care services. The study found that the permanent increase in income reduced the use of institutional care services by the older adults (4). Luo and Ding, based on the data from the 2005–2018 China older adult health survey, found that increasing the income of the older adults played an important role in promoting the utilization of institutional care services (2).

Two papers are particularly relevant to this paper. Goda et al. used a natural experiment to estimate the impact of endowment insurance income on the utilization of long-term care services (4). The study found that the increase in endowment insurance income led to a decline in the utilization of institutional care services, but an increase in the utilization of formal family care services. The evidence showed that formal family care services replaced institutional care services. Wang studied the impact of social security income in China on the use of formal care services for the older adults, and found that as the retirement income of the older adult increases, the older adult tend to use formal care services rather than family care services provided by their children (19). Different from the above two literatures, this paper focuses only on the utilization of institutional care services, and takes the disabled older adults living in cities and towns as the research objective to explore the impact of pension benefits on the utilization of institutional care services. In addition, the paper further discusses the differences in the impact of pension benefits on the utilization of institutional care services for the older adults with disabilities across income levels and whether the increased pension benefits reduce the support burden on the children of the disabled older adults.

### 2.2 Theoretical analysis

There is some debate in existing researches about whether institutional care service for the older adults is a normal good or an inferior good. In economics, the distinction between normal and inferior goods is generally made based on the income elasticity of demand. The income elasticity of demand for normal goods is greater than 0, the income elasticity of demand for luxury goods is greater than 1, and the income elasticity of demand for necessities is greater than 0 and less than 1; the income elasticity of demand for inferior goods is less than 0. Nyman estimated that the income elasticity of institutional care services was about 1.2, and believed that institutional care services were normal goods, which was equivalent to the elasticity of general medical services (24). Some studies also suggest that institutional care services are inferior goods. Charles and Sevak found that for every $10,000 increase in annual income, the probability of nursing home use decreased by only 1% (25). Goda et al. found that for every $1,000 increase in annual social security income, the likelihood of nursing home use decreased by 2 to 3 percent, and that the decrease in the use of institutional care services was due to substitution by home care services (4). Study has also argued that China’s institutional care services have a “luxury” nature, as the level of fees exceeds the ability of the older adults to pay (26). However, no empirical evidence has been found to support the view that institutional care services are “luxury goods”.

In this paper, we use a family decision model, following Pezzin et al., which considers household utility as a function of daily goods, leisure, care services, and household preferences (7). Suppose that in a family with only one disabled older adult, except for the consumption of care services, the daily consumption of the family is a fixed value, and the utility function of the choice of care services depends on the combination of different types of care services. Therefore, a simple utility function is as follows:


(1) $$U=F\left(X_{F},X_{N},\;I\right)$$

**Figure 1:**
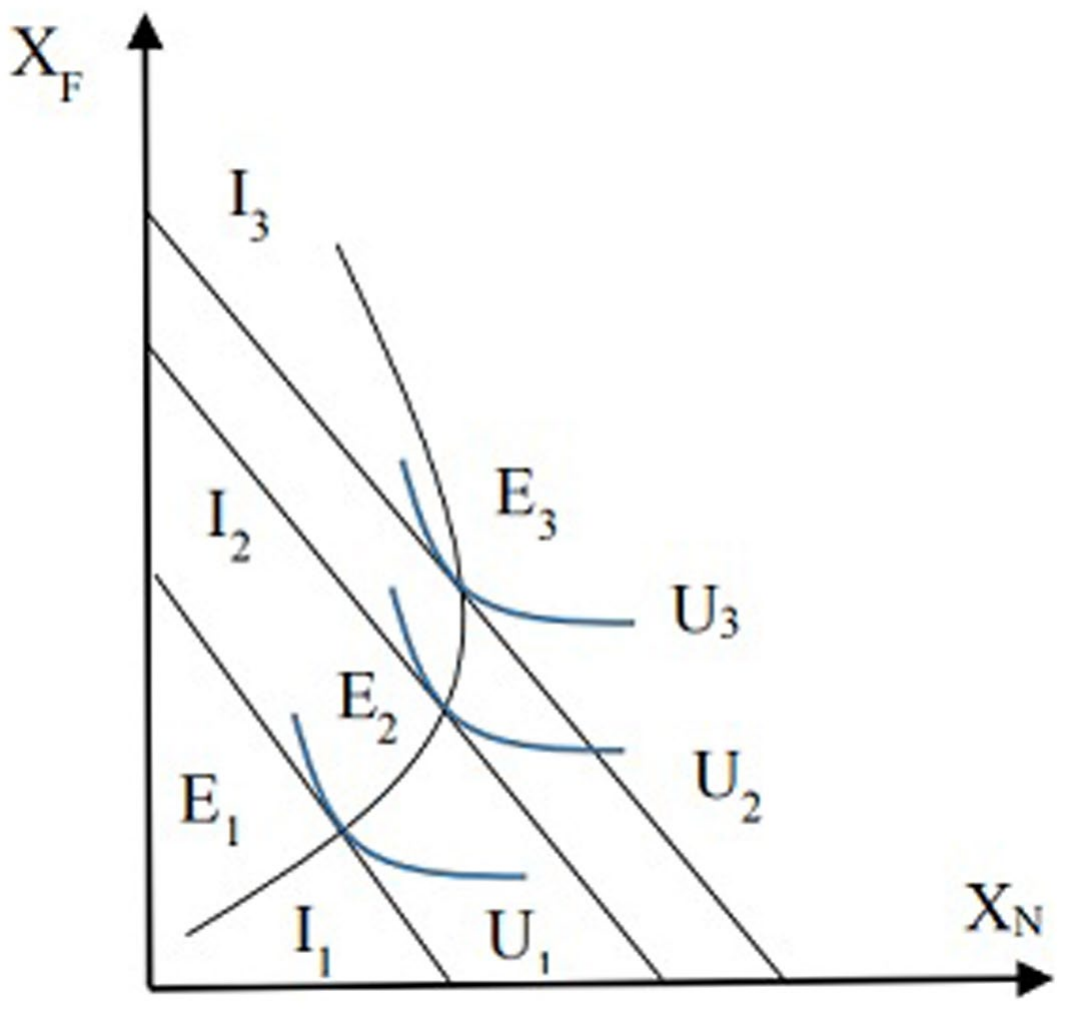
The income - consumption curve of care services for the older adult.

Among them, I represents the income of the older adult, and X_F_ represents non-institutional care services, including informal home care services, formal home care services, community care services, etc., X_N_ is the demand for institutional care services, and non-institutional care services and institutional care services are completely substituted. Under budget constraints, utility maximization becomes the best combination of different types of care services. When the pension is constantly changing, the curve of the optimal consumption mix of care services for the disabled older adult can be shown in Figure 1.

In Figure 1, the vertical axis represents the number of older adult’ demand for non-institutional care services X_F_, and the horizontal axis represents the number of demand for institutional care services X_N_. The consumption budget lines for the older adult are represented by I_1_, I_2_, and I_3_, and U_1_, U_2_, U_3_ is the undifferentiated curves, while E_1_, E_2_, and E_3_ display the optimal consumption combinations of institutional and non-institutional care services. As can be seen in Figure 1, from point E_1_ to point E_2_, as pension benefits increase, the use of both institutional and non-institutional care services by the older adult increases, thus both of them are normal goods. But when income increases to a certain extent, such as from point E_2_ to point E_3_, with the increase in income, the utilization of institutional care services decreases, at this time, institutional care services become low-grade goods. Therefore, whether a good is low-grade or normal is related to the level of income, and for the older adults with different income levels, institutional care services may belong to normal goods or low-grade goods. The impact of pension benefits on the use of institutional care services may be different for disabled older adults at different income levels. Thus, the following hypotheses are proposed in this paper:

*Hypothesis 1*: An increase in pension benefits improves the utilization probability of institutional pension services for the disabled older adult.

*Hypothesis 2*: The effect of pension benefits on the utilization of institutional care services for the urban disabled older adults varies according to income differences.

## 3 Methods

### 3.1 Equation

Since the utilization of institutional care services for the older adult is a dummy variable, this paper uses a probit model to estimate the effect of pension benefits on the utilization of institutional care services for the disabled older adult. The model is constructed as follows:


(2) $$\begin{array}{c} \mathrm{uliin}s_{ip}=\beta_{0}+\beta_{1}\log\left(\mathrm{pensio}n_{ip}\right)+\beta_{2}\mathrm{perso}n_{ip}\\ +\beta_{3}\mathrm{famil}y_{ip}+FE_{p}+\varepsilon \end{array}$$

$\mathrm{uliin}s_{ip}$ is the utilization of the institutional care services of an older adult, $\beta_{1}$ is the regression coefficient. $\mathrm{perso}n_{ip}$ and $\mathrm{famil}y_{ip}$ represent the personal and family characteristic variables of the older adult, and the regression coefficients are $\beta_{2}$ and $\beta_{3}$ , respectively. $FE_{p}$ is the province fixed effect, and ε is the random variable disturbance term.

The utilization of institutional care services for the disabled older adults is also influenced by the financial status of their children. The worse the economic status of the children, the less likely it is that a disabled older adult will use institutional care services. Due to data limitations, variables such as children’s income are not controlled for in this paper. Therefore, this paper uses instrumental variables to address the problem of omitted variables. Following the idea of constructing grouped means as instrumental variables proposed by Fisman and Svensson, this paper also constructs instrumental variable based on three exogenous variables grouped by age, education, and region, and the mean of the pension within the group is used as the instrumental variable (27).

We use stata16 statistical software to perform descriptive and regression analyses of the data and model.

### 3.2 Data and variables

The data in this paper is from the 2017–2018 Chinese Longitudinal Health Longevity Survey. It is a tracking survey of the older adults conducted by the Center for Healthy Aging and Development Studies of Peking University. The survey area covers 23 provinces (municipalities or autonomous regions) across the country, and the respondents are older adults aged 65 and above and adult children aged 35–64. The questionnaire includes two types: one for surviving respondents and another for family members of deceased older adults. After a baseline survey in 1998, the survey program conducts follow-up surveys in 2000, 2002, 2005, 2008–2009, 2011–2012, 2014, and 2017–2018, with the most recent follow-up survey (2017–2018) interviewing a total of 15,874 individuals aged 65 and older.

According to the research needs, this paper uses the questionnaire of the surviving older adults in 2017–2018, and selects the sample according to the following requirements: (a) the older adults who currently live in cities and towns. According to the questionnaire: the category of the current residence of the respondents: 1. city 2. town 3. rural. We select the older adults who currently live in cities and towns. (b) the older adults with impaired ability of daily life. According to the responses of the older adults to the six activities of daily living in the questionnaire, those who do not need help in all six activities of daily living are considered to be self-sufficient older adults, otherwise, they are considered to be disabled older adults. A total of 2,515 samples of urban disabled older adults over the age of 65 are finally obtained.

The explanatory variable is the utilization of institutional care services for the older adult. According to the question in the CLHLS questionnaire: With whom do you currently live: 1. with family (including a caregiver); 2. alone; 3. in an older adult care institution. According to the survey data, 81.31%, 7.64%, and 11.04% of the older adults live with family members, live alone, and live in older adult care institutions, respectively. In this paper, the older adults who answered the option3 are assigned a value of 1, which means that they used the institutional care services; the older adults who answered the options 1. and 2 are assigned a value of 0, which means that they did not use the institutional care services.

The core explanatory variable is the monthly pension benefits. In the questionnaire, the following question is asked for those who are entitled to the retirement system, “If you have left/retired, what is your monthly pension?.” For older adults who are not covered by the retirement system, the following question is asked “what is the monthly pension you receive now?.” We set the pension benefits variable based on these two questions.

The control variables include individual characteristics, family characteristics, and province-fixed effects. Specifically, the following variables are introduced for the individual characteristics: the gender variable, which takes the value 1 for men and 0 for women; the age variable, which is the chronological age of the respondent at the time of the interview. Considering the possible non-linear relationship between the use of institutional care services and age, the quadratic term of age is also introduced; the ethnic variable: the value of Han nationality is 1, and the value of minority nationality is 0; Number of years of education, is the actual number of years of schooling for the older adult; Marital status, according to the questionnaire question:Your current marital status is: 1. married and living with your spouse, 2. married but not living with your spouse, 3. divorced, 4. widowed, and 5. never married. The response of option 1 is assigned 1 and the others are assigned 0; Disability level, is measured based on respondents’ self-assessment of six daily activities, including bathing, dressing, toileting, controlling urination, indoor activities, and eating. For example, in the questionnaire question: Do you need help when taking a shower (including wiping your upper or lower body): 1. No help needed, 2. A specific area needs help, 3. Two or more parts need help, the answer of option1 is assigned a value of 0, otherwise it is assigned a value of 1. We use the sum of the six daily activities to measure the level of disability; Observed health, is obtained from the interviewer’s record of observation of the older adults’ health status in the questionnaire, according to the question: The older respondent looked: 1. surprisingly healthy 2. relatively healthy 3. moderately ill 4. very ill. We assign a value of 4 to the older adults who answered option 1, a value of 3 to those who answered option 2, a value of 2 to those who answered option 3, and a value of 1 to those who answered option 4; The main source of income, according to the questionnaire: What is your main source of income now? 1. pension 2. spouse 3. children 4. grandchildren 5. other relatives 6. local government or association 7. own work or job 8. Other. We assign a value of 1 to the answers option1, option2 and option7, which represent the economic independence of the older adults. We assign a value of 2 to the responses of option3, option4, option5 and option8, indicating that the older adults are supported by their children, and a value of 3 to the answer of local government or association, indicating that the older adults are socially supported by the government.

The family characteristic variables include the following variables: number of children, which is the number of surviving children of the older adult respondent; total annual family income, which is the total annual income of the entire family of the older adult respondent in the last year.

In addition, the study controls for province-fixed effects, to control for unmeasured province-level factors that affect both monthly pension and utilization of institutional care services.

Table 1 shows the descriptive statistical results of the main variables of urban disabled older adult. According to the survey data of CLHLS2017-2018, there are 2,515 disabled older adult over 65 years old, of which 11% lived in care institutions. 1,470 samples receive pension income, and the average pension is 2873.94 yuan/month. In terms of personal characteristics of the disabled older adults, male disabled older adults account for 35%, with an average of 2.68 years of education. 97% of the sample were of Han nationality. 17% of the older adults were married and their partners were alive. On average, there were about 3 activities of daily living that required assistance. In terms of health level, 7.9% of the disabled older adults were very ill and 34.2% were moderately ill, 50.6% were relatively healthy and 7.3% were surprisingly healthy. 39.5% of the disabled older adults were financially independent, 50.5% were supported by their children, and 10% were supported by government social assistance. The average total annual income of disabled older adult families was about 50,227.98 yuan, and the average number of children was 3.67.

**Table 1:** Descriptive statistics.

Panel A	*N*	Mean (SD)	Median(Q1,Q3)	Min, max
Monthly pension	1,470	2,873.94 (2,686.02)	2,600 (400, 4,000)	80, 10,500
Age	2,515	94.73 (8.62)	97 (90, 101)	65, 116
Years of education	2,281	2.68 (4.28)	0 (0, 5)	0, 20
Disability level	2,515	3.11 (1.93)	3 (1, 5)	1, 6
Family income	2,285	50,227.98 (37,040.05)	49,200 (13,000, 100,000)	99, 100,000
Number of children	2,417	3.67 (1.82)	4 (2,5)	0, 11
**Gender**				
0.female	1,636 (65.0%)			
1.male	879 (35.0%)			
**Ethnic**				
0.others	61 (2.6%)			
1.han	2,270 (97.4%)			
**Marry**				
0. Divorced, widowed or unmarried	2,046 (82.5%)			
1.married	434 (17.5%)			
**Observed Health**				
1. very ill	197 (7.9%)			
2. moderately ill	853 (34.2%)			
3. relatively healthy	1,263 (50.6%)			
4.surprisingly healthy	182 (7.3%)			
**Main source of financial support**				
1. Self-support	906 (39.5%)			
2.Children or other relative	1,158 (50.5%)			
3.local government or community	230 (10.0%)			

The data are from the 2017–2018 Chinese Longitudinal Healthy Longevity Survey.

In Table 2, we also analyze the utilization of institutional care services by the disabled older adults in the higher and lower pension benefits groups^1^. In the higher pension benefits group, 13.4% of the disabled older adults were living in institutions, and only 6.7% living in institutions in the lower group. A value of p less than 0.001 indicates a significant difference between the two, which is also a preliminary test of the hypothesis 1. In addition, we also divide the disabled older adults into low-income and high-income groups based on their family income, and analyze the utilization of pension benefits and institutional care services within different groups. In groups with different income levels, the utilization rate of institutional care services for older adults with higher pension benefits is higher than that of disabled older adults with lower pension benefits. However, the value of p shows that this difference is significant within the low-income group, while not significant within the high-income group, which is consistent with hypothesis 2.

**Table 2:** Descriptive testing of hypotheses.

Utilization of institutional care Services	Total	High-pension benefits^a^	Low-pension benefits	*p* value
	N = 2,515	N = 1,637	N = 878	<0.001
0.No	2,214 (89.0%)	1,400 (86.6%)	814 (93.3%)	
1.Yes	274 (11.0%)	216 (13.4%)	58 (6.7%)	
Low-income family^b^	N = 1,143	N = 813	N = 330	<0.001
0.No	1,011 (89.3%)	702 (87.2%)	309 (94.5%)	
1.Yes	121 (10.7%)	103 (12.8%)	18 (5.5%)	
High-income family	N = 1,372	N = 778	N = 594	0.133
0.No	1,203 (88.7%)	670 (87.6%)	533 (90.2%)	
1.Yes	153 (11.3%)	95 (12.4%)	58 (9.8%)	

a Grouping based on the average pension benefits within the group.

b Grouping based on the median of the family income.

## 4 Empirical results

### 4.1 Baseline estimation results

Table 3 reports the results of estimating the effect of pension benefits on the utilization of institutional care services for the urban disabled older adults. Column 1 reports the regression equation without the addition of control variables and the marginal effects of the estimation results. The results show that pension benefits are significantly and positively associated with the probability of using institutional care services for the urban disabled older adults. Column 2 controls for individual characteristic variables of the disabled older adults based on the regression equation in column 1. The results show that higher pensions significantly increase the probability of using institutional care services for the disabled older adults. Column 3 additionally controls for household characteristic variables based on column 2, and the coefficient on pensions stays positive at the 1% level of significance. Column 4 controls for province-fixed effects based on column 3. The results of the regression indicate that an increase in pensions considerably contributes to an increase in the probability of using institutional care services for the disabled older adults. On average, a 1% increase in pensions is associated with a 3 % increase in the probability of using institutional care services for the disabled older adults.

**Table 3:** Impact of pensions on the utilization of institutional care services.

	(1)	(2)	(3)	(4)
Monthly pension	0.04***	0.07***	0.07***	0.03*
	(0.01)	(0.01)	(0.01)	(0.02)
age		−0.01	−0.00	−0.00
		(0.02)	(0.02)	(0.02)
Square term of age		−0.00	−0.00	−0.00
		(0.00)	(0.00)	(0.00)
Years of education		−0.00	−0.00	−0.00
		(0.00)	(0.00)	(0.00)
Gender		−0.02	−0.01	0.01
		(0.02)	(0.02)	(0.02)
Ethnic		0.01	−0.01	−0.02
		(0.06)	(0.06)	(0.07)
Marital		−0.11***	−0.11***	−0.12***
		(0.03)	(0.03)	(0.03)
Observed health		−0.01	−0.01	0.00
		(0.01)	(0.01)	(0.02)
Disability level		0.03***	0.03***	0.03***
		(0.01)	(0.01)	(0.01)
**Main source of income**				
Children support		0.08*	0.04	0.03
		(0.04)	(0.04)	(0.04)
Government support		0.08*	0.05	0.05
		(0.05)	(0.04)	(0.04)
Annual household income			−0.03***	−0.03***
			(0.01)	(0.01)
Number of children			−0.02***	−0.02***
			(0.01)	(0.01)
Individual characteristics	NO	YES	YES	YES
Family characteristics	NO	NO	YES	YES
FE	No	No	No	YES
Pseudo-R^2^	0.039	0.172	0.202	0.254
Observations	1,460	1,171	1,126	875

(1) the regression coefficient reported in this table is the marginal effect; (2) Standard errors in parentheses; (3) * * * *p* < 0.01, **p < 0.05, *p < 0.1.

Among the control variables, the probability of using institutional care services is lower for the married disabled older adults than for the unmarried, divorced, or widowed disabled older adults. This could be due to the fact that, compared to unmarried, divorced, or widowed disabled older adults, married older adults have access to family care from their spouses or children, resulting in a reduced probability of opting for institutionalized care services. As the degree of disability increases, so does the utilization of institutional care services by older adults in urban areas. A study discovered that as the degree of disability increases, the probability of a disabled older adult choosing nusing care institutions increases (28). Additionally, the number of children has a substantial unfavorable impact on the utilization of institutional care services by urban residents. As the number of children increases, the probability that urban residents will choose institutional care services decreases because more home care can be provided. There is a significant negative correlation between total family income and institutional care services, with higher-income families being more financially strong and thus more likely to choose home care services, ultimately reducing the probability of institutional care services.

### 4.2 Robustness test

To further test the regression results, we conduct additional tests for robustness as follows.

#### 4.2.1 Instrumental variable estimation

Pension benefits may have potential endogenous issues. Pension benefits may crowd out private savings and intergenerational economic transfers, etc., resulting in a negative correlation with the personal wealth of the older adult (4, 19, 20). If more accurate personal wealth information of the older adult is not available, the impact of pension benefits on the utilization of institutional care services may be underestimated, because the older adult with more sources of income is more likely to use formal care services. To address the endogeneity problem, this study employs an instrumental variable, the mean pension value within the group, for estimation.

Table 4 presents the instrumental variable estimation results. Columns 1 and 2 demonstrate the iv-probit estimation outcomes, which suggest that the coefficient on the average value of pensions within-group is positive at the 1% level of significance. This suggests that the average value of pensions within-group significantly explains pension benefits. Meanwhile, the AR test statistic is 3.42 and significant at the 10% level, while the Wald test statistic is 3.15 and also significant at the 10% level, indicating that there is no weak instrumental variable problem. The results from the second-stage regression demonstrate that pension benefits have a notable and positive impact on the utilization of institutional care services for the disabled older adults, indicating that the endogeneity problem does not alter the conclusions of this paper. The 2SLS regression results are provided in columns 3 and 4. The F-statistic value in the first stage is 29.749, surpassing the critical value of 10, indicating the absence of a weak instrumental variable problem. The regression results from the second stage show that the impact of pension benefits on institutional care services for older adults with disabilities is strong.

**Table 4:** Instrumental variables estimator.

	(1)	(2)	(3)	(4)
	First stage	Second stage	First stage	Second stage
Average value of pensions within group	0.40***		0.37***	
	(0.75)		(0.07)	
Monthly pension		0.84***		0.14**
		(0.29)		(0.06)
Individual characteristics	YES	YES	YES	YES
Family characteristics	YES	YES	YES	YES
FE	YES	YES	YES	YES
Observations	875	875	1,126	1,126
Test statistics	AR test Statistic	3.42*	1st stage F statistic	29.749
	Wald test Statistic	3.15*		

(1) the regression coefficient reported in this table is the marginal effect; (2) Standard errors in parentheses; (3) * * * *p* < 0.01, **p < 0.05, *p < 0.1.

#### 4.2.2 Sample adjustment

This paper’s samples are disabled older adults. The definition of disabled older adult was derived from responses to the questionnaire, which assessed six daily living activities, including bathing, dressing, toileting, controlling defecation and urination, indoor activities, and eating. Based on another question in the survey questionnaire: Have you been restricted in your daily activities in the past 6 months due to health issues? 1. Yes, there are significant limitations. 2 Yes, to some extent restricted.3 There are no restrictions. We redefine the disabled older adult for robustness testing. We define those who answered option 3 as not disabled and assigned a value of 0, otherwise they were disabled and assigned a value of 1, and then re-select the sample of disabled older adults accordingly. Column 1 of Table 5 reports the regression results after redefining the sample of disabled older adults, which is consistent with the baseline regression. In addition, those who answered option 2 may not use institutional care services because of the low level of disability. Therefore, we define the older adults who responded to option 1 as disabled. Column 2 reports the results of the regression, and we find no significant differences from the baseline regression.

**Table 5:** Robustness test (probit).

	(1)	(2)	(3)	(4)
Monthly pension	0.03**	0.05**	0.03*	0.03*
	(0.01)	(0.02)	(0.02)	(0.02)
Individual characteristics	YES	YES	YES	YES
Family characteristics	YES	YES	YES	YES
FE	YES	YES	YES	YES
Pseudo-*R*^2^	0.247	0.305	0.254	0.256
Observations	1,162	401	875	875

(1) The regression coefficient reported in this table is the marginal effect; (2) Standard errors in parentheses; (3) * * * *p* < 0.01, **p < 0.05, *p < 0.1.

Widowed and single older adult women may be more likely to reside in nursing homes due to the lack of family care support. The inclusion of these women in the sample may result in biased estimation results (20). To address this concern, we re-estimated the regression model after excluding widowed and single older adult women. The regression result is reported in column 3 of Table 5. We find that the conclusion aligns with the baseline regression, supporting the reliability of the findings.

#### 4.2.3 Add control variables

In addition, this paper tests the resilience of a variable that has a lasting effect on the health condition of the older adult, namely, participation in medical insurance (20). The corresponding results are presented in column 4 of Table 5, and they align with the baseline regression.

### 4.3 Heterogeneity analysis

The utilization of institutional care services for urban older adult is mainly driven by economic and social characteristics (1). Studies have shown that older adults with lower educational attainment (29) and females (30) are more inclined to utilize older adult services. Therefore, is there heterogeneity in the utilization of institutional care by the disabled older adults?

Columns 1 and 2 of Table 6 report the effect of pension on the utilization of institutional care services for male and female disabled older persons, respectively. The findings demonstrate that increased pensions significantly enhance the probability of using institutional care services for male disabled older adults, but have no significant effect on female disabled older adults. This could be a result of female older adults in China relying more on their families than males (31). Columns 3 and 4 of Table 6 report the effect of pension on the use of institutional care services by financially independent and financially dependent disabled older adults. The results show that pension benefits statistically increase the probability of utilizing institutional care services for the financially dependent disabled older adults, but have no significant effect on the financially independent older adults. Columns 5 and 6 in Table 6 report the impact of pension benefits on the utilization of institutional care services for the older adults living in cities and towns, respectively, and we find that the effect of higher pensions on the probability of using institutional care services is significantly positive for the disabled older adults living in towns, but there is no significant effect for the disabled older adults living in cities. Possible reason for this is that the financially dependent older adults and the older adults living in towns and villages are low-income groups for whom institutional care service is a “luxury,” so the positive effect of pension benefits is greater. In contrast, the disabled older adults who are financially independent and live in urban areas have higher income levels and are more likely to use home care services.

**Table 6:** Heterogeneity analysis.

	(1)	(2)	(3)	(4)	(5)	(6)
	male	female	financially independent	financially dependent	city	town
Monthly pension	0.05*	0.02	0.02	0.04*	0.01	0.03*
	(0.03)	(0.02)	(0.02)	(0.02)	(0.02)	(0.02)
Individual characteristics	YES	YES	YES	YES	YES	YES
Family characteristics	YES	YES	YES	YES	YES	YES
FE	YES	YES	YES	YES	YES	YES
Pseudo-*R*^2^	0.230	0.298	0.221	0.464	0.247	0.337
Observations	329	404	589	216	547	201

(1) the regression coefficient reported in this table is the marginal effect; (2) Standard errors in parentheses; (3) * * * *p* < 0.01, **p < 0.05, *p < 0.1.

## 5 Discussion

### 5.1 Difference in different income groups

In this paper, the estimated coefficient β1 is 0.03 in the benchmark regression, which indicates that a 1% increase in pension benefits results in a 3 percentage points increase in the probability of using institutional care for the older adults. But our estimate of β_1_ = d(*uliins*) /d (log *pension*) is not equal to the income elasticity, and can only reflect the magnitude of the income elasticity to some extent. According to the income elasticity formula, the income elasticity of utilization of institutional care services = d (*uliins*)/ (*uliins*) /d (log *pension*) = β_1_/*uliins*. We calculate the average probability of utilization of institutional care services for the disabled older adults, which is 0.15. We then calculate the income elasticity of utilization of institutional care services for the disabled older adults, which is 0.2 (0.03/0.15 = 0.2). This suggests that institutional care service is a normal good and that the demand for institutional care services would increase when income increases. Since we cannot calculate the amount of demand for institutional care services, the elasticity here is not equal to the income elasticity of demand, but it can reflect the trend of the size of the income elasticity of demand to a certain extent, which in turn helps us to preliminarily judge the commodity nature of institutional care service. Therefore, we determine whether institutional care service is a normal good or a luxury good for the disabled older adults based on the magnitude of the income elasticity of utilization of institutional care service, which has been done in the existing literature, such as Goda et al. (4), Wang (19) and Tsai (20). We consider institutional care service to be a normal good if the income elasticity of utilization of institutional care service is greater than 0 and less than 1, and a luxury good if the income elasticity is greater than 1.

Since the income elasticity of utilization of institutional care service is greater than 0 and less than 1, so we consider the institutional care service is a normal good for the disabled older adults. However, the probability of utilization of institutional care service increases with changes in pension benefits, and the income elasticity of utilization of institutional care service declines naturally with increasing pension benefits. This is also in general agreement with the findings of existing studies. The income elasticity of demand for a good may change from high to low, or even become negative, as income increases, maintaining prices and other conditions constant (32). In this paper, we categorize the older adults with disabilities into two groups, low-income and high-income, according to the median family income, and test whether the effect of pension benefits on the utilization of institutional care service for the older adults with disabilities varies according to differences in the older adults’ income.

Columns 1 and 2 of Table 7 present how pension benefits affect the utilization of institutional care services among older adults with disabilities across various income levels. The findings demonstrate a positive but insignificant correlation between pension benefits and utilization of institutional care services among high-income disabled older adults, while low-income disabled older adults exhibit a positive correlation at a significance level of 5%. Specifically, a 1% increase in pension benefits increases the probability of institutional care utilization by low-income disabled older adults by 0.06. The income elasticity of utilization of institutional care services for low-income disabled older adults is calculated to be 0.29(0.06/0.21); the income elasticity of utilization of institutional care services for high-income disabled older adults is calculated to be 0.13 (0.02/0.15). It suggests that the utilization of institutional care services for low-income urban disabled older adults is more sensitive, i.e., more elastic, to changes in pension benefits. Compared to high-income older adults, when the income of low-income disabled older adult increases, the demand for institutional care services increases more.

**Table 7:** Difference in different income groups and utilization of substitutes.

	(1)	(2)	(3)	(4)
	Low-income	High-income	Low-income	High-income
Explanatory variable	Institutional care service	Institutional care service	Home care service	Home care service
Monthly pension	0.06**	0.02	−0.08**	0.03
	(0.03)	(0.02)	(0.03)	(0.02)
Individual characteristics	YES	YES	YES	YES
Family characteristics	YES	YES	YES	YES
FE	YES	YES	YES	YES
Pseudo-*R*^2^	0.401	0.204	0.183	0.080
Observations	245	520	339	571

(1) the regression coefficient reported in this table is the marginal effect; (2) Standard errors in parentheses; (3) * * * *p* < 0.01, **p < 0.05, *p < 0.1.

### 5.2 Difference in the utilization of substitutes

Substitutes for institutional care services include home care services and informal home care. There is more evidence, both theoretical and empirical, that institutional care services and home care services, as paid formal care services, are substitutes for home care (4, 19, 20). Regarding the substitution between institutional and home care services, Goda et al. found that the increase in social security income led to the substitution of home care services for institutional care services (4). In this paper, we consider only the effect of pension benefits on the choice of formal care services for the disabled older adult, i.e., the substitution relationship between formal home care services and institutional care services.

Based on the questionnaire question: What was the total cost of these care payments (e.g., direct costs such as labor, goods, etc.) for nearly a week? We set the variable formal home care service utilization.Disabled older adults who have paid for care services but are not living in an institution are assigned a value of 1 to indicate that they have used formal home care services, otherwise they are assigned a value of 0 to indicate that they have not used formal home care services. Columns 3 and 4 of Table 7 present the impact of pension benefits on the utilization of formal home care services by older adults with disabilities at various income levels. The findings indicate a significant negative correlation between pension benefits and the use of home care services for low-income disabled older adults, as well as an insignificant positive correlation for high-income disabled older adults. This suggests that for low-income disabled older people, the utilization of paid home care services by low-income disabled older people has declined significantly as pension benefits have increased.

### 5.3 Pension benefits and the burden on children

Researches have shown that disability increases the cost of family healthcare for older adults (33), and over 30% of disabled older adults require financial support from relatives (34). In China, children continue to be the primary caregivers for most older adults with disabilities (35). It can be seen that the children of disabled older adults may face both economic and care pressures at the same time. The increase in pension benefits can, to some extent, lessen the caregiving burden for children by allowing for greater use of institutional care services for disabled older adults. Additionally, by increasing the financial independence of disabled older adults, there is a higher chance that they will purchase institutional care services using their own funds, which in turn reduces the financial burden on their children. The paper further explores the impact of pension benefits on the burden on adult children providing eldercare.

Columns 1–3 of Table 8 report the impact of pension benefits on the provision of care services by children, son and daughter-in-law, and daughter and son-in-law, respectively. The findings indicate a notable decrease in the probability of children providing care services to the disabled older adults, and a notable decrease in the probability of sons and daughters-in-law providing care services, but no significant change in the probability of daughters and sons-in-law providing care services as the pension benefits increase. This indicates that the pension benefits notably lessen the care responsibility of children, particularly sons and daughters-in-law. This could be attributed to the reality that sons and daughters-in-law are currently the primary providers of family caregiving (36, 37), and male family members are gradually abandoning the older adult support responsibilities as a result of higher pension benefits (19), which making the impact of pension benefits more significant for this demographic. Column 4 of Table 8 presents the effect of pension benefits on whether children pay for the older adult disabled. The findings reveal that pension benefits significantly decrease the probability that children pay for care, suggesting that pension benefits reduce the financial burden on children of the disabled older adult.

**Table 8:** Utilization of institutional care services and burden on children.

	(1)	(2)	(3)	(4)
Explanatory variable	Care from children	Care from sons and daughters-in-law	Care from daughters and sons-in-law	Pay by children
Monthly pension	−0.04**	−0.04**	0.01	−0.03*
	(0.02)	(0.02)	(0.02)	(0.02)
Individual characteristics	YES	YES	YES	YES
Family characteristics	YES	YES	YES	YES
FE	YES	YES	YES	YES
Pseudo-R^2^	0.247	0.143	0.080	0.293
Observations	1,055	1,114	1,125	925

(1) the regression coefficient reported in this table is the marginal effect; (2) Standard errors in parentheses; (3) * * * *p* < 0.01, **p < 0.05, *p < 0.1.

## 6 Conclusion

Income is an important determinant of utilization of institutional care services for the disabled older adults. Research on whether an increase in pension benefits encourages the use of institutional care services for disabled older adults is inconclusive. In this study, we use data from the 2017–2018 CLHLS to examine the impact of pension benefits on the utilization of institutional care services for the urban older adults with disabilities. The research shows that an average 1% increase in pension benefits raises the probability of utilization of institutional care services among urban older adults by 0.03. The effect is significant for disabled older adults who are male, financial dependent, and living in towns. This is because older women are more dependent on their families, their utilization of institutional care services is not sensitive to changes in pension benefits. However, older adults who are financially dependent or reside in towns have lower income levels, and the utilization of institutional care services is more sensitive to the changes in pension benefits. Further study finds that institutional care services are normal goods for urban disabled older adults, while the low-income disabled older adults are more sensitive to them. Higher pension benefits reduce the burden on children by reducing the probability of children providing care at home, especially sons and daughters-in-law, and reducing the probability of children paying for care.

The conclusions of this paper are similar to the existing research. Du and Wang found that the utilization of social care services has a phenomenon of “pro-high income,” which is more obvious in the utilization of rural social care services (1). Wang studied the impact of social security income on the utilization of nursing services and found that as social security income increased, the older adults were more inclined to use social nursing services, while male family members provided fewer home nursing services (19). In contrast to existing research, the research objective of this paper focuses on the utilization of institutional care services for disabled older adults in cities and towns. We find that the higher the pension benefits, the higher the probability of using institutional care services for disabled older adults, but this is more significant for low-income older adults.

Under the dual pressure of the aggravation of population aging and childlessness in China, the shortage of nursing services is a serious challenge faced by the nursing system for the disabled older adults. Especially for low-income disabled older adults, family members are the main service providers, and only a small number of older adults can enjoy home based community care services and institutional care services (38). Therefore, there is an urgent need to comprehensively promote the personal pension system and improve the multi-level insurance system to improve the pension benefits for low-income disabled older adults, which will help transform this potential care demand into real needs. Second, there is an urgent need to speed up the full coverage of long-term care insurance, reduce spending on care services for the disabled older adults, and reduce the burden of older adult care on the families of the disabled older adults. Thirdly, for towns and rural areas with lower income levels, it is necessary to provide more care services and eliminate the imbalance in urban–rural supply.

This paper explores the utilization of institutional care services as whether the older adults use institutional care services or not. In terms of specific types, institutional care services include life care services, medical and health services, spiritual comfort services, and so on. However, this paper does not explore the specific types of the older adults’ use of institutional care services in depth, which is a limitation of this paper. In addition, this paper uses the composite variable “disability level” to reflect the degree of disability of the older adults, and ignores the number and severity of illnesses suffered by the older adults, which are important limitations of the study. In addition, as older adults age and become more disabled, their likelihood of participating in family decision-making decreases, and their utilization of institutional care services is more likely to be determined by their children or spouses (39). We did not explore this issue in the theoretical analysis section, which is also worth paying close attention to in the future. Using the family decision-making model, our future research direction focuses on the impact of pension benefits on other decision-making behaviors of disabled older adults, such as the use of home care services, the use of medical services, and so on.

## Data availability statement

Publicly available datasets were analyzed in this study. This data can be found at: https://opendata.pku.edu.cn/.

## Ethics statement

Ethical review and approval was not required for the study on human participants in accordance with the local legislation and institutional requirements. The patients/participants provided their written informed consent to participate in the survey.

## Author contributions

XX: Writing-original draft, Writing-review & editing, Methodology. QL: Conceptualization, Formal Analysis.

## Footnotes

^1^Here, the higher group and the lower group are classified according to the average pension benefit of the disabled older adults, and those whose pension benefit is lower than the average are placed in the lower group, otherwise they are placed in the higher group.
